# Non-linguistic Conditions for Causativization as a Linguistic Attractor

**DOI:** 10.3389/fpsyg.2017.02356

**Published:** 2018-01-23

**Authors:** Johanna Nichols

**Affiliations:** ^1^Department of Slavic Languages and Literatures, University of California, Berkeley, Berkeley, CA, United States; ^2^Linguistic Convergence Laboratory, Higher School of Economics, National Research University, Moscow, Russia; ^3^Faculty of Arts, University of Helsinki, Helsinki, Finland

**Keywords:** verb, causative, language spread, mixed language, selection, attractor, linguistic symbiosis, linguistic frontier conditions

## Abstract

An attractor, in complex systems theory, is any state that is more easily or more often entered or acquired than departed or lost; attractor states therefore accumulate more members than non-attractors, other things being equal. In the context of language evolution, linguistic attractors include sounds, forms, and grammatical structures that are prone to be selected when sociolinguistics and language contact make it possible for speakers to choose between competing forms. The reasons why an element is an attractor are linguistic (auditory salience, ease of processing, paradigm structure, etc.), but the factors that make selection possible and propagate selected items through the speech community are non-linguistic. This paper uses the consonants in personal pronouns to show what makes for an attractor and how selection and diffusion work, then presents a survey of several language families and areas showing that the derivational morphology of pairs of verbs like *fear* and *frighten*, or Turkish *korkmak* ‘fear, be afraid’ and *korkutmak* ‘frighten, scare’, or Finnish *istua* ‘sit’ and *istutta* ‘seat (someone)’, or Spanish *sentarse* ‘sit down’ and *sentar* ‘seat (someone)’ is susceptible to selection. Specifically, the Turkish and Finnish pattern, where ‘seat’ is derived from ‘sit’ by addition of a suffix—is an attractor and a favored target of selection. This selection occurs chiefly in sociolinguistic contexts of what is defined here as linguistic symbiosis, where languages mingle in speech, which in turn is favored by certain demographic, sociocultural, and environmental factors here termed frontier conditions. Evidence is surveyed from northern Eurasia, the Caucasus, North and Central America, and the Pacific and from both modern and ancient languages to raise the hypothesis that frontier conditions and symbiosis favor causativization.

## Introduction

Sociolinguistics and social context change languages. By now it is understood that absorption of an appreciable number of L2 speakers eventually leads to decomplexification of the absorbing language (the spreading one in a language shift) (Trudgill, 2011), mass bilingualism beginning in childhood can complexify languages (ibid., Dahl, 2004), dense and closed social networks retard language change while open ones foster it (Milroy and Milroy, 1985, 1992), differential degrees of social connection favor uptake and transmission of innovations (Fagyal et al., 2010), and a language whose speakers have less reliable access to vital resources is likely to have more variation and its speakers to be more accepting of variation than one whose speakers are more secure (Hill, 2001a). But what kinds of (non)-complexity, what kinds of changes, and what kinds of variation?

Here I deal with a specific effect of sociolinguistics on grammar and in particular on grammatical categories: a type of sociolinguistic situation described below appears to favor selection of attractors. An attractor, as the term is understood in complex systems theory, is any state that is easier to enter or acquire than to leave or lose, and/or easier to retain than lose. Selection refers to both uptake and transmission, so that in the end selected features expand in frequency and range, diffusing through both the grammar and the speech community.

**Figure 1:**
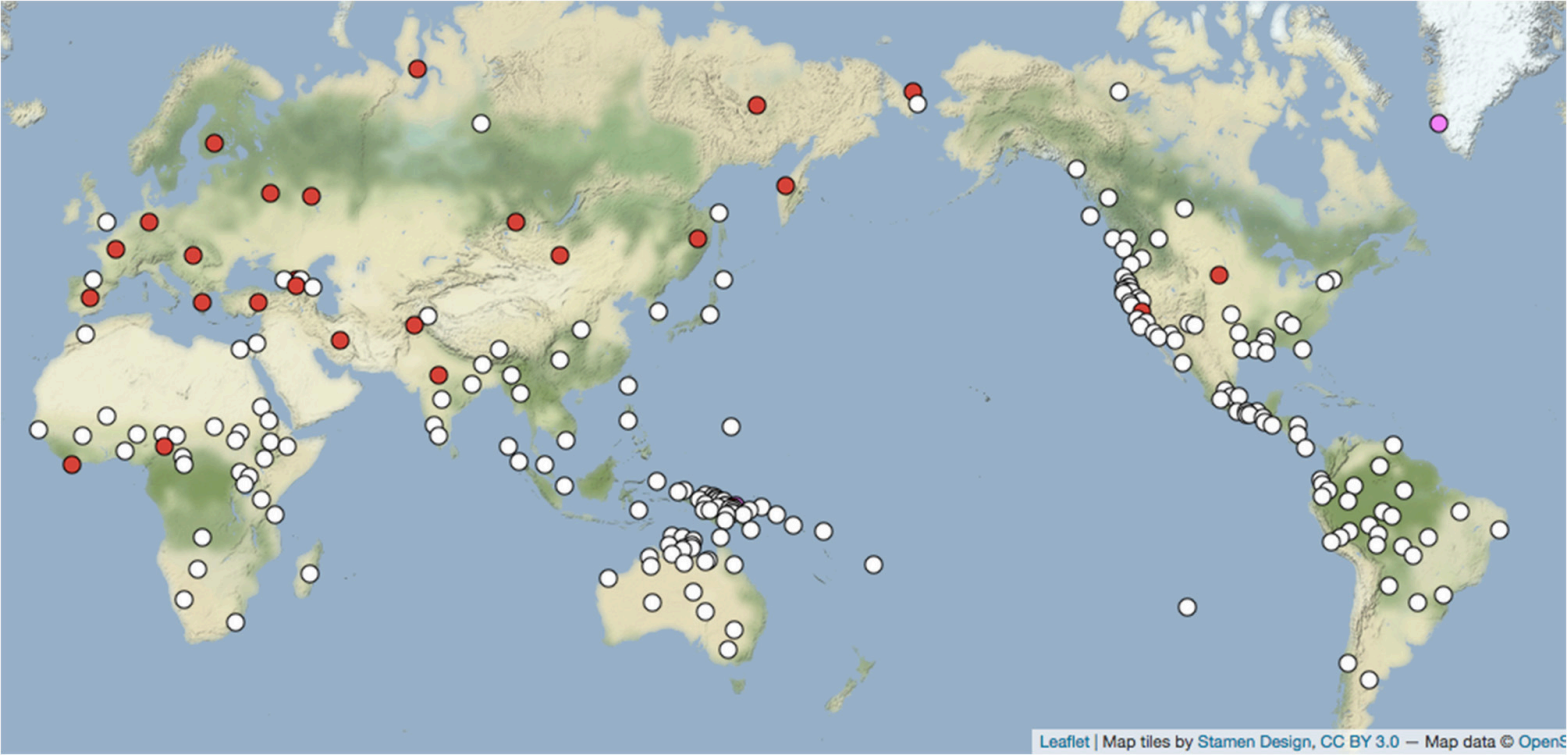
*m-T* pronoun paradigms (*N* = 230). Red = *m-T* paradigm present; white = absent (Nichols and Peterson, 2013a,b). http://wals.info/feature/136A#2/24.8/153.6.

**Figure 2:**
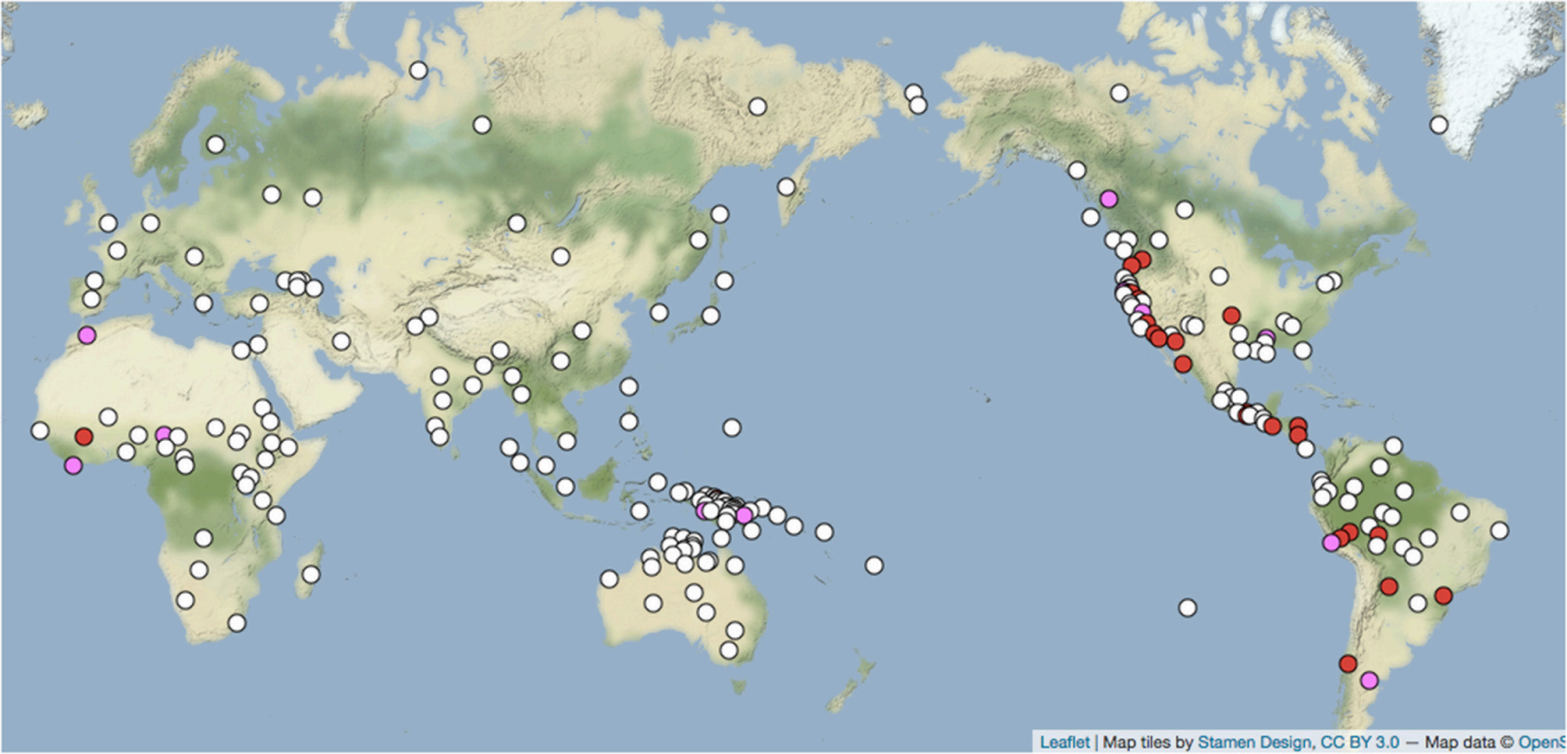
*n-m* pronouns (*N* = 230). Red = *n-m* paradigm present; white = absent; pink = non-paradigm present (Nichols and Peterson, 2013a,b). http://wals.info/feature/137A#2/24.8/153.6.

Figures 1, 2 (Nichols and Peterson, 2013a,b) show a known attractor that can serve as an introductory example: the phoneme /m/ figuring in personal pronoun systems with a counterposed anterior consonant such as /t/, /č/, /s/, henceforth symbolized with a generic T. Examples are Finnish *minä* ‘I’ and *sinä* ‘you’ [and similarly for most of the the sister Uralic languages of Finnish, e.g., Erzya Mordvin (central Russia) *mon, ton*, Selkup (Samoyedic branch, southern Siberia) *man, tan*], Georgian (Kartvelian family) *me, šen*, Latin *me* ‘me’ (accusative case), *te* ‘you’ (accusative), and many others. This pattern of first person /m/ and second person T is widespread in northern Eurasia, where it occurs in several separate language families and in most daughters of those families, but it is quite rare elsewhere (Figure 1). A similar pattern occurs in the western Americas, where a number of languages have first person /n/ and second person /m/, e.g., Wintu (Wintun, northern California) *ni* ‘I, we’, *mi* ‘you’; Pipil (Uto-Aztecan, Nicaragua) *nu*- ‘my’, *mu*- ‘your’; Mapudungun (isolate, Chile) ñ*i* ‘my’, *mi* ‘your’ (Figure 2). Each of these patterns is frequent and densely attested in several separate language families in its own macrocontinent, but very rare elsewhere.

This geography indicates that each system has enjoyed an evolutionary advantage in its respective macrocontinent—and only there. Furthermore, the Eurasian pattern, where we have longer historical records and early attestation of languages, has demonstrably expanded over the last few millennia (Nichols, 2012a,b, 2013), with gains outnumbering losses and /m/ in particular sometimes gained in pronouns but almost never lost from them. Now, /m/, /n/, and T (especially in the form /t/) are very basic sounds, learned early by children, present in the sound systems of most languages, and easily audible, but if these factors motivated their expansion and stability in pronouns we would expect *m-T* pronoun systems to be common worldwide. Pronouns are rarely borrowed from one language to another, and abstract consonantal skeletons of words are borrowed very rarely if at all; but these factors have not inhibited spreads of pronoun forms in Eurasia and the Americas. Thus, what calls for explanation here is the distribution revealed by the geography.

What appears to favor the emergence and spread of such systems is, first, attractor status and second, a sociolinguistics that favors selection of attractors even in the resistant domain of pronouns. Why these systems are attractors is not covered here, and in any case accounting for it would amount to explaining frequencies of elements in the pre-existing variation that selection works on, rather than describing the mechanism of selection itself (to put it in terms of Darwinian theory). Here, evidently the status of attractor becomes relevant, and selection goes to work, only in the presence of factors other than the phonetic basicness of /m/ or the grammatical basicness of pronouns. Those factors appear to center on ones enhancing the prospects for emergence and uptake of attractors.

Of those factors the one studied here is a sociolinguistics I call *linguistic symbiosis* because it involves two (or more) languages functioning as a single communicative system while remaining discrete (i.e., without forming a mixed language). Symbiosis is the essential coexistence of, and possibility of selection from, more than one language variety, where both (or all) varieties are neutrally valued, selection is bidirectional (or multidirectional), and code switching is accepted. Less technically, in symbiosis two (or more) languages function side-by-side in a society under conditions that make it possible for the languages mingle in speech and for the speakers to select from both languages in a single utterance. The extent and frequency of such mingling are much greater than in ordinary code switching–as, for example, if in discussing Peruvian cuisine I insert the term *aji amarillo* (a variety of pepper), complete with Spanish phonology, into an English sentence or perhaps put the entire phrase or sentence containing it into Spanish (as is possible and not uncommon if both the interlocutor and I know Spanish well). Symbiotic intermingling, in contrast, may be so thoroughgoing that it is difficult for a linguist to decide which of the languages an utterance is in, though the languages actually remain discrete (i.e., they do not merge to create a single mixed language, as occasionally happens under somewhat different sociolinguistic conditions: see e.g., Bakker and Mous, 1994; Meakins, 2013). The main sociolinguistic conditions that make symbiosis possible are lack of a standard or prestige language (which might favor use of one language over the other), minimal or absent language identity or other ideology linking language to other aspects of identity, acceptance of code switching, and sufficient dialect or language diversity to offer a range of options to choose from. Examples are discussed below. This sociolinguistic context facilitates selection and in particular lets attractors be selected because they are attractors and not (e.g.) because they are emblematic of a prestige language.

Propagation of selected attractors is another matter. It is evidently favored by factors that provide opportunities for lateral transmission: sufficiently dense social networks, sufficient distant social connections, and sufficient population mobility, to maintain connections and expose individuals to linguistic diversity, including the range of variation made possible by code switching and bilingualism; and sufficient population density to make possible numerous and long-range social connections and repeat contacts with the same individuals or groups. Some level of density, extent, and reliability of contacts makes it possible for some individuals to be well-connected, and this seems to be essential to the uptake and transmission of innovations (Fagyal et al., 2010). Now, sufficient population density to suppport dense and extensive social networks, in ecological and economic conditions supportive of mobility, has probably existed to any appreciable extent only since the rise of food production. It is no accident that the *m-T* pronoun systems are thickly attested among the language families that have been involved with the rise and spread of nomadic pastoralism in Eurasia, where population growth, long-range client-patron and guest-host connections, and mobility were hallmarks of the societies and essential to the spreads of their languages (see Anthony, 2007; Nichols and Rhodes, 2017). Prehistoric sociolinguistics is difficult to determine, but in the surviving fossil of the frontier of the Eurasian pastoral expansion, Khamnigan Mongol (Janhunen, 1990, 1991, 2005; Yu, 2011), there is easy code switching and apparently little language identity, though the languages remain discrete and the mingling of forms in speech does not lead to language shift or mixed languages.

Linguistic symbiosis must have been an important part of the sociological and ethnic situations that have obtained at the frontiers of large language spread like those reviewed below, where new economic and social opportunities are constantly being created and and an enterprising individual can seize or create a new niche. In such undertakings clear communication is essential and the means of communication can be improvised. This situation is what Nichols and Rhodes (2017) call *frontier* conditions: an interface involving cultural, economic, and technological intermingling and offering prospects for entrepreneurship, intermediary roles, and trade management distant from the center of authority and prestige.

Sometimes a society at the frontier has taken advantage of this situation and its members have seized the roles of merchant, tinker, interpreter, diplomat, mercenary, camp follower, money changer, organizer, and/or others who mediate between the expanding culture and those beyond the frontier. Sometimes the frontier society melts into the expanding one, but sometimes its language spreads out far in advance of the expanding one. This is a *catalyst language* (Nichols and Rhodes, 2017), so called because the intermediary roles of its speakers assist or make possible the spread of the expanding language; examples include Ainu (catalyst for the Japanese Yayoi expansion), Tungusic (catalyst for the Mongolic northeastward expansion), the Mongolic family itself (catalyst for the northward expansion of Chinese empire), and several Turkic expansions (catalyst for the westward expansion of Chinese economic control) (see Janhunen, 2002, 2008, 2012). These languages have spread far from their points of origin (Ainu survived only at its own far northern frontier). These languages all bear markers of attractor spread, and it seems likely that symbiosis is a regular trait of catalyst languages.

Linguistic symbiosis overlaps in part with what Hill (2001a) calls a *distributed stance:* an outlook or attitude that tolerates variation on the part of others and generates variation in the speaker's own output. Its development is favored by contingent or unreliable access to vital resources and a combination of mobility and sparse population that causes individuals to grow up without a stable cohort of age mates and thus without a dialect identity. Hill's examples come from desert populations, where resource insecurity and high mobility are the rule. I count the elements of the distributed stance (contingent access to resources, weak or no dialect identity) as factors that contribute to symbiosis, together with the sociolinguistic properties identified here (diversity, neutral valuation of varieties). These are distinct from the factors discussed above that stabilize selected variants: dense networks, long connections, open connections, and any others that favor uptake and transmission of what would otherwise be one-off selections.

Factors that can be symptomatic of symbiosis where we have no direct evidence include archaeological, economic, and/or political-historical evidence for back-and-forth shifting of cultural or economic or political allegiance, bidirectional pattern copying (calquing, grammatical borrowing, etc.) in languages, direct or indirect evidence of catalyst function, and large-scale expansion of a language in a desert or high-latitude environment. Below these are used as operative criteria for positing prehistoric symbiosis. The Khamnigan Mongol case mentioned above is important because it preserves linguistic symbiosis at what was the frontier of Mongol economic and linguistic expansion. It shows that identifying spreads of a certain type with symbiosis is a safe move.

## Method and survey

The case for pronoun consonantism rests on an attractor that becomes relevant and selection that becomes operative only in the right sociolinguistic and demographic context. Though the geography supporting this account is compelling, it is circumstantial evidence. Actually testing the claims is problematic because pronoun consonantism is difficult to work with statistically: the range of options is small, essentially just ‘yes’ vs. ‘no’ (i.e., /m/ or no /m/) per language and per pronominal category; conforming languages are a minority even in those continents where they are most frequent; the pronominal context is defined as a search through options (independent pronouns, verb agreement affixes, possessive affixes, etc.), the generic T for the Eurasian second person is also a set of options, and casting about through options inflates the possibility of success. Therefore, what follows seeks evidence of sociolinguistic and sociological conditioning in a more tractable part of grammar: the causative alternation.

The causative alternation is illustrated in the verb pairs shown in Table 1. Each pair consists of a non-causal verb (‘laugh’, ‘die’, ‘sit’, etc.) and the corresponding causal (‘make laugh’, ‘kill’, ‘seat’, etc.), whose semantics consists of the non-causal predicate plus causation: ‘frighten, scare’ means ‘cause to fear or be afraid’ and causal (transitive) ‘break’ means ‘cause to break or get broken’. The semantic relationship of non-causal and causal is alike for each pair, but the formal structures differ, and the point at issue here is how, grammatically and structurally, the two verbs in each pair are related. The causal can be derived from the non-causal, as in Estonian ‘fear’: ‘frighten’ or Kazakh ‘break’; the non-causal can be derived as in Macedonian ‘fear’ or Czech and Spanish ‘break’; both can be derived, as in Aymara ‘break’; completely different verbs can be used, as in Norwegian, Catalan, and Russian ‘fear’: ‘scare’; or the two forms can be identical as in German ‘break’ (and English *break* and many other verbs).

**Table 1:** Some causal/non-causal verb pairs.

	**Non-causal**	**Causal**
	‘fear, be afraid’	‘frighten, scare’
Macedonian	**se** plaši	plaši
Russian	bojat'-**sja**	pugat'
Estonian	hirmuma	hirmu-**ta**-ma
Norwegian	frykte	skremme
Catalan	témer, tenir por	espantar, esporuguir
	‘break’ (intransitive)	‘break’ (transitive)
Czech	lomit **se**	lomit
Spanish	romper-**se**	romper
Aymara	p'aki-**si**-	p'aki-**ñ**a-
Kazakh	synu	syn-**dyru**
German	brechen	brechen

*The suffix or other morphology deriving one from the other is boldface. Hyphens are for clarity (they are not orthographic in the languages)*.

I used a set of 18 such pairs (Nichols et al., 2004), assembled from dictionaries and/or consultation with native speakers and language experts, surveyed across 207 languages, about half of which figure centrally here. The pairs are listed in Table 2. Most of the counts and graphs below use only the nine such pairs that typically have an animate undergoer (e.g., ‘fear’, ‘angry’, ‘sit’), as these tend to be more stable over time.

**Table 2:** Surveyed causal-noncausal verb pairs.

**Animate**		**Inanimate**	
**Non-causal**	**Causal**	**Non-causal**	**Causal**
laugh	make laugh, amuse	(come to) boil	(bring to) boil
die	kill	burn, catch fire	burn, set afire
sit	seat	break	break
eat	feed	open	open
learn	teach	be/get dry	dry (off, out)
see	show	straight	straighten (out)
be/get angry	anger, make angry	hang, dangle	hang (up)
fear, afraid	scare, frighten	turn over	turn over
hide	hide	fall	drop

*Animate, inanimate = typically undergone by animate or inanimate entity*.

**Figure 3:**
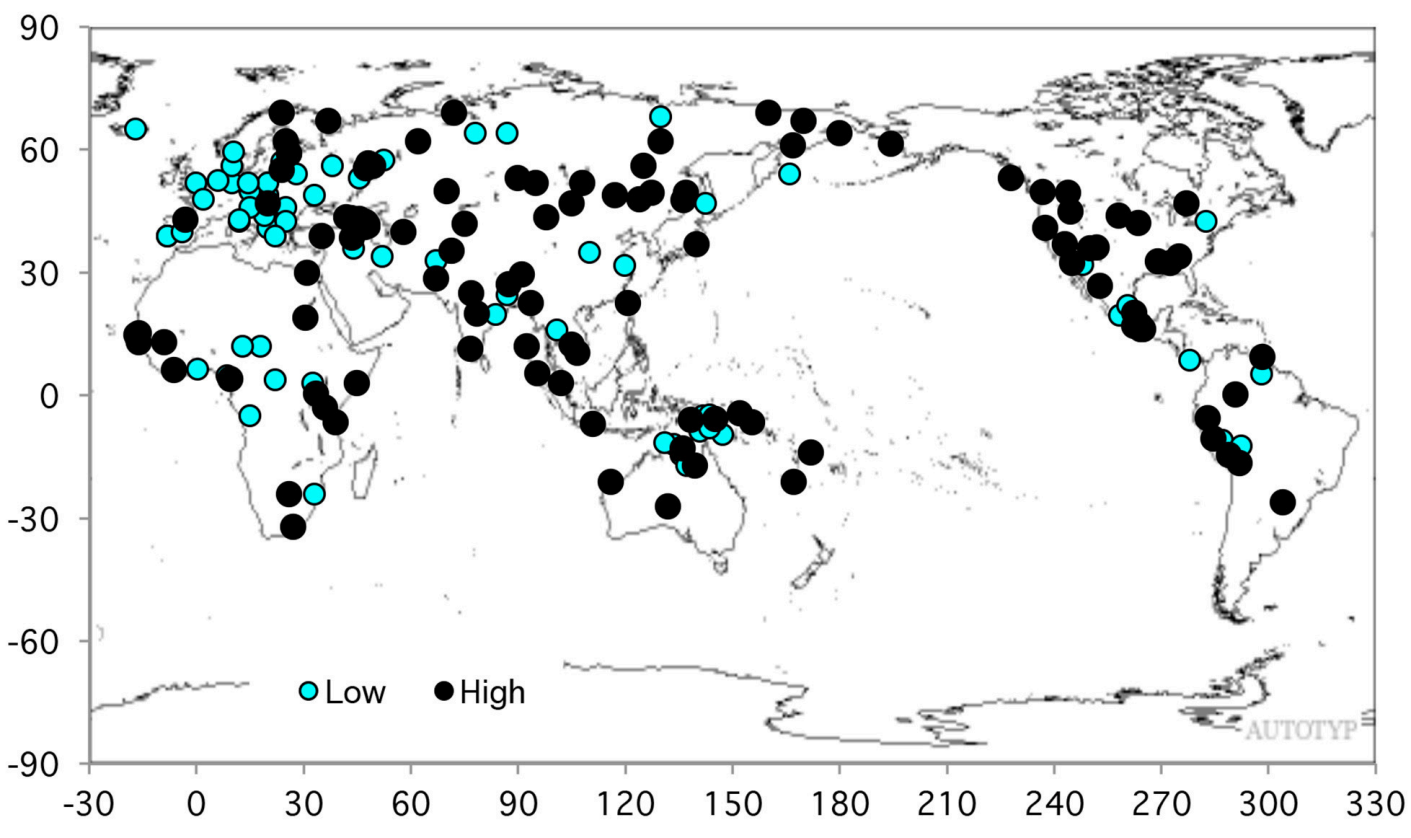
Distribution of high and low frequencies of causativization in the 9-pair verb list (*N* = 200). Black = above mean; blue = below mean (Mean = 54%).

The formal relationships between the two members of the pair can be reduced to three basics: the causal form is derived; the non-causal is derived; they have the same vs. different roots. Languages are typologized by the percent of the pairs exhibiting those three basic types, and what primarily figures here is the percent that use causativization, i.e., derivation of the causal from the non-causal (as in Estonian ‘fear: scare’ and Kazakh ‘break’ in Table 1). Of interest here is preferred causativization, i.e., above-mean percentage of pairs in which the causal is derived. (The mean for the animate set of verbs is 54%, or just under five pairs.) As shown in Figure 3, high and low percentages are not evenly distributed worldwide: very few pairs use causativization in Europe (blue symbols) and many in northern Asia and North America. (A sparser but essentially similar picture emerges if they are plotted as ±1 standard deviation from the mean). What predominates in Europe is decausativization, where the non-causal is derived from the causal, as in Spanish *romperse* ‘break’, Macedonian *se plaši*, and others. (In these two examples the verbs are reflexive, a derivational type that is common in Europe but infrequent elsewhere).

The hypothesis here is that, of the possible realizations of the causative alternation, causativization is an attractor that is selected in symbiosis. There seem to be two reasons why causativization is an attractor. First, it aligns with semantics. In a verb pair like ‘sit’ and ‘seat’, ‘sit’ involves only a subject and an activity or position, while ‘seat’ adds an agent and semantics of causation. If ‘seat’ is derived from ‘sit’, the morphological form echoes the semantics and the cognitive complexity^1^. Second, most languages have a ready source of potential causativizing morphemes: verbs like ‘make’ function easily to create phrases with causative semantics (e.g., *That always makes me laugh*, where *make laugh* is well on the way to being lexicalized as a discontinuous causative verb). In very many languages the causativizing morphology is in fact a reduced form of a verb like ‘make’ that has become a causativizing suffix. There is no comparably ready source for decausativization, which involves removal of the agent and the agency. Reflexive pronouns derive non-causatives in many European languages, but this is an idiosyncratic construction, not common outside of Europe, with no correlation to the semantics: ‘get angry’ may look literally like ‘make oneself angry’, but that is not at all the meaning.

That causativization is associated with symbiosis was first suggested (not using the term *symbiosis*, and describing the sociolinguistics differently), in Nichols (2011). Here I draw on expanded data, improved coding, and improved understanding of the sociolinguistics (Grünthal and Nichols, in press) to give firmer results from more parts of the world. Despite these advances, this is a hypothesis-raising study, using a database originally designed for other purposes, which uses a standard sampling approach that strives for independence of languages by choosing only one per family or major branch, while what is needed for hypothesis testing is dense coverage of families. The goal here is to determine whether such further testing would be worthwhile.

## Results

This section reports seven case studies supporting an association of causativization with symbiosis and/or frontier conditions.

### The northeastern caucasus

The first case study is what I call the Avar sphere in the eastern Caucasus, from the middle ages to the Russian conquest of the Caucasus in the mid nineteenth century. It involves mostly protohistorical and early historical spreads and a sociolinguistic situation that was viable until the mid-twentieth century and is still in evidence, so we are on firm ground in describing it^2^. At the time of the conquest the Avar khanate dominated the north slope of the eastern Caucasus (a.k.a. Daghestan). The Avar khanate was the continuation of the Sarir Kingdom, which arose c. 800 BCE (and changed its name to Avar on converting to Islam)^3^. Prior to the Russian conquest, the Avar khanate was an economic and cultural power and a military confederation encompassing a large number of small highland and foothill city-states located along highland watercourses, chiefly the Avar Koisu and Andi Koisu rivers, their tributaries, and their lowland confluence in the Sulak (which flows to the Caspian Sea), whence there were connections to Silk Road ports and cities. The city-states were independent and could join or leave the confederacy at will; mostly they joined and remained, and while they were members their young men served in the Avar army, where Avar served as language of command. For millennia, since the adoption of food production, Daghestanian highland societies were half transhumant, with the working-age male population spending the winter half of the year in the lowlands tending herds in winter pastures and/or taking seasonal employment or owning businesses in lowland cities. The non-transhumant female part of the population traveled downhill regularly to the market towns or the larger lowland markets. Roads ran along river canyons, so the Avar Koisu and Andi Koisu roads funneled all such traffic to the confluence, where the Avar capital Khunzakh was strategically located in an ideal position for trade and taxation.

For these essential economic contacts highlanders needed to know foothill and lowland languages, but not vice versa; lowlanders had no need to travel uphill, rarely did so, and did not learn highland languages. As a result, the linguistic situation in Daghestan involved massive local asymmetrical vertical bilingualism and multilingualism with an overlay of Avar as an always available contact language. In mountain areas, languages generally spread uphill from the economically better-connected and more densely populated lowlands to the more isolated highlands, and the vertical bilingualism of Daghestan strengthened that tendency: Avar or the language of a market town, known to many people from towns above it, could come to be used in a higher town as well. The language family spoken in most of the eastern Caucasus is Nakh-Daghestanian, an old and much-differentiated family, and as a result of repeated uphill spreading the daughter branches, most of which are of about Romance-like or Slavic-like diversity and apparent age (so ~2,000 years), extend from lowlands to the highest inhabited levels. The archaeological age of villages, where known, is generally well over 2,000 years. This gives reason to reconstruct repeat uphill spreads of Nakh-Daghestanian branch ancestors, probably accompanying periods of economic prosperity in the lowlands, ever since the Nakh-Daghestanian dispersal several millennia ago.

The Avar language is now spoken along the Sulak, all along the Avar Koisu and beyond, spilling over the crest to northern Azerbaijan, and along the lower Andi Koisu with occasional outliers in the highlands. Those are outliers of lowland northern dialects, so the Avar dialect diversity there is not great. The diversity of Avar along the Avar Koisu is greater, but still all dialects are said to be more or less mutually intelligible. This suggests that the Avar spread began some 500 years ago; more than about 500 years generally spells loss of ready mutual intelligibility (of course all such figures are very approximate). The Andic subbranch, the closest sister to Avar, extends above Avar along the Andi Koisu, with two outliers along the Avar Koisu. The Andic languages are closely related but generally not mutually intelligible, and this together with the more uphill position indicates that the Andic uphill spread was somewhat earlier than the Avar one. Andic place names are found Avar lands along the lower Andi Koisu and the Sulak, testifying to Avar expansion there. The Andi, a large foothill Andic group for whose language the branch is named, were economically powerful until the Russian conquest, collecting taxes along the Andi Koisu and into the Chechen lands in the west, and monopolizing the lucrative trade in the Caucasian *burka*, the felt coat worn by highland shepherds and the czarist Russian army. The Andi and Avar were rivals for political and economic power (the Andi won their last important battle against the Avars in the late seventeenth century).

The more distantly related Tsezic languages are uphill of the Andic ones along the Andi Koisu, with two outliers on upper tributaries to the Avar Koisu. They evidently represent a still earlier spread, whose lower languages have since shifted to Andic much as Andic has shifted to Avar in the lowlands.

Repeated uphill spreads would mean absorption of highland populations by language shift, with adults learning the spreading language. This should bring about decomplexification of the language, and indeed Avar and the Andic languages show considerable decomplexification compared to most other Nakh-Daghestanian languages. In addition, however, there is evidence of linguistic symbiosis. The mix of spreads described above implies oscillating dominance of Avar and ancestral Andic, depending on political and economic fortunes in the lowlands. In addition, there was no standard language and no source of linguistic prestige apart from market and inter-ethnic usefulness (to the extent that any language was prestigious it was Arabic, and that only after the conversion to Islam). Another factor favoring linguistic symbiosis was the mobility of the transhumant societies. In addition, there was little or no language ideology or identity; the foci of identity were clan, village, and in recent centuries sometimes religion. Social networks were dense but open, with many long-range contacts both uphill and downhill. The pan-Daghestanian term for a host in a guest-host relationship is *kunak*, and such connections were sought and valued, especially at long distances or when they involved a well-placed lowlander.

The interaction of Avar with local Andic and Tsezic languages was a historically documented matter of symbiosis, with free interjection of Avar words into the local language. Some such words have by now stabilized as loans but some are one-time code switching. e.g., in Hinuq (Tsezic), Avar adjectives “constitute an open class in the sense that whenever a Hinuq speaker wants to use an adjective and does not find a Hinuq term (s)he uses an Avar term” (Forker, 2013, p. 170). Pronouns in Avar, Andic, and Tsezic languages are strongly assonant, using both rhyme and alliteration, much of it innovative compared to Proto-Nakh-Daghestanian (Nichols, 2012b): examples are in Table 3.

**Table 3:** Avar-Andic-Tsezic pronouns.

	**1sg**	**2sg**	**Inclusive**	**1pl**	**2pl**
Avar	dun	mun	nił	niž	nuž
Godoberi (Andic)	den	min	ié	išše	bitté
Hunzib (Tsezic)	de	me		ile	miže

*Nominative case only. 1sg = first person singular, 2pl = second person plural, etc*.

These languages make extensive use of causativization. Table 4 shows Avar verb pairs from the list above (Creissels, 2014). Table 5 shows percentages of causativization in the Avar sphere and elsewhere in the Caucasus. Percentages decrease with distance from Avar, both within the Avar sphere and between groups. The languages shown cover the Avar sphere, other languages of the eastern Caucasus (Lak, Dargwa, Lezgi, Tsakhur, etc.), and languages to the west (Chechen, Ingush), and they include languages on both the north and south slopes. The conclusion is that, where symbiosis has been most common, causativization is most frequent. No other known factor accounts for the frequency of causativization within Nakh-Daghestan.

**Table 4:** Avar verb pairs.

‘laugh’, ‘make laugh’	beł-ize	beł-iz-**ab**-ize
‘sit’, ‘seat’	k'us-ize	k'us-iz-**ab**-ize
‘eat’, ‘feed’	k'wan-aze	k'wan-az-**ab**-ize
‘see’, ‘show’	bix-ize	bix-iz-**ab**-ize
‘get angry’, ‘make angry’	ccin+daxx-ine	ccin+daxx-in-**ab**-ize
‘fear’, ‘frighten’	- hinq'-ize	- hinq'-iz-**ab**-ize
‘hide’	baxč-ize	baxč-ize;
		baxč-iz-**ab**-ize

*Causativizing suffix bold. Hyphens (not orthographic) segment off the infinitive ending and the causative suffixx*.

**Table 5:** Eastern and central Caucasus: Proportion of the nine verb pairs that use causativization.

Avar sphere:	Avar	0.72
	Akhvakh	0.89
	Karata	0.89
	Bagwalal	0.78
	Godoberi	0.89
	Tsez	0.78
	Hinuq	0.67
	Hunzib	0.50
Nearby:	Lak	0.50
	Dargwa	0.67
	Chechen	0.67
Distant:	Ingush	0.56
	Archi	0.39
	Udi	0.56
	Tabassaran	0.25
	Rutul	0.11
	Tsakhur	0.25
	Lezgi	0.56

*Languages are listed within groups in order of increasing distance from the Avar capital (in the Avar sphere this amounts to increasing altitude). Hunzib is peripheral to the Avar sphere; its winter pastures and other connections were in Georgia to the south*.

### The eastern steppe

The eastern, or Mongolian, steppe is the band of grassland extending along the south slope of the southern Siberian mountains from the Tien-shan and Altai to north central China^4^. Here, from the rise of mining and metallurgy in the Altai area and the rise of imperial power in China, successive nomadic pastoral tribes, kingdoms, and states have formed and their languages have spread far, chiefly westward, along the steppe and in Central Asia. The spreads have generally involved conquest of rulers and language shift by much of the population, with the result that the languages that have undergone large spreads are considerably decomplexified and regularized in their grammars and lexicons. There has also been a good deal of borrowing and grammatical convergence among them: the modern Turkic, Mongolic, and Tungusic languages in particular are strikingly similar in their overall structures. In historical and protohistorical times the various expansions have created frontier conditions along the expanding periphery, and there is firm evidence of linguistic symbiosis in the surviving Khamnigan Mongol-Evenki situation mentioned above. Zgusta (2015, pp.104–164) gives evidence of frequent movement and realignment of ethnic groups along the lower Amur that appear likely to have involved symbiosis among different Tungusic languages and with unknown pre-Tungusic languages.

The known language families involved in these spreads, in chronological order of earliest importance, are eastern Iranian (Indo-European), Turkic, and Mongolic. Other, poorly attested languages are likely to have been involved in the early stages, perhaps including an ancestral Yeniseian language (the family is historically attested only along the upper and middle Yenisei, with Ket on the middle Yenisei the only survivor). The medieval and later Turkic and Mongolic spreads are historically and ethnographically well described and some of the sociolinguistics is attested or reconstructable. The two families both originated in or near today's northern Mongolia and seem to have had connections to both the Altai metallurgical center and the steppe nomadic economies. Between these two families, locally and in general along the frontier, there was some history of back-and-forth shifting, each functioning as catalyst to the other at least some of the time. Before the rise of Genghis Khan Mongolic was spreading at westward and absorbing Turkic speakers (Janhunen, 2008). During the Mongol expansion, Turkic speakers whose tribes and states had been incorporated into the Mongol empire were so much more numerous than Mongols that, although Mongolian was the language of command, it was Turkic rather than Mongolic speech that was chiefly spread across Central Asia and the central and western steppe.

The nomadic pastoral economy, which propelled the spreads, fostered mobility and contacts with other peoples and languages around the steppe periphery: hunter-gatherers in the north who traded in furs; miners and metalworkers in the Altai area; urban centers in China and Central Asia; various trade outposts. Language identity among nomads appears not to have been strong, and there were no standard or written languages and no durable prestige language. Clan and client-patron relations were primary. In addition to the decomplexification and regularization that testifies to histories of language shift, the languages of both families and also the neighboring Tungusic family to the east have pronoun systems with rhyme, alliteration, and the *m-T* type that bespeak symbiosis. Causativization is high overall, highest in Turkic, which has the longest history of nomadic spreading, and least high in Tungusic, a family of languages spoken by settled semi-agriculturalists in northern China and Korea and spread in Siberia by reindeer herders (Table 6). Within each family, languages closest to the center of symbiosis have the highest percentages, supporting the correlation of symbiosis with causativization.

**Table 6:** The Turko-Mongol steppe and neighbor Tungusic: Proportion of the nine verb pairs that use causativization.

Turkic	Tatar	0.88
	Kazakh	1.00
	Turkmen	1.00
	Chuvash	1.00
	Yakut	0.88
	Uighur	0.75
	Kirgiz	0.89
	Tuvan	0.88
	Khakas	0.67
Mongolic	Khalkha	0.75
	Buriat	1.00
	Dagur^*^	0.67 (only six pairs found)
	Khamnigan^*^	0.67 (only three pairs found)
Tungusic	Manchu	0.88
	Nanai	0.78
	Udehe	0.83
	Evenki	0.63
	Even	0.50

*Within each family, languages closer (or historically closer) to the centers of expansion are listed first*.

* *Proportions not accurate as not all of the nine pairs could be found*.

### Uralic

The Uralic family stretches across northwestern and north central Eurasia, from western Norway beyond the Yenisei to the eastern Taimyr Peninsula, a distribution that was continuous down to about the southern limit of the northern forest zone until the relatively recent northward expansions of the Scandinavian languages and Russian^5^. Testifying to its long presence in the region and the momentum of its spread, the family has representatives in the three linguistically diverse accretion zones to the south of its main range: the eastern Circum-Baltic area (Estonian and several small languages), the middle Volga (Erzya and Moksha Mordvin, Mari), and south central Siberia (Samoyedic languages in the Altai mountains, now extinct). These zones are populated by remnant languages from other prehistoric spreads. Most of the westward spread of Uralic postdates, and was probably triggered by, the Indo-Iranian expansion c. 4,000 years ago from what is now northeastern Kazakhstan (a number of early Iranian or Indo-Iranian loans entered the Proto-Finno-Ugric branch of Uralic at that time). The westernmost extension—the spread of Finnic into Finland and Saami into Scandinavia—occurred less than 1,000 years ago, before which first ancestral Saami, then early Finnic, had been adopted by agricultural people in the east Baltic area (probably Germanic- and Baltic-speaking; both of these are Indo-European branches). Spreads of North Saami within Saami (in Scandinavia) and Nenets within Samoyedic (in Siberia) are also recent and involved the spread of reindeer herding.

These spreads were at high latitudes and involved sparse and mobile populations (even the agriculturalists of southern Finland were relatively mobile and sparse, relying on slash-and-burn methods and moving to new fields from time to time). The known large spreads—Saami, Finnic, Tundra Nenets—can therefore be assumed to have involved symbiosis, and it is these large spreading languages that have the highest proportions of causativization (Table 7), supporting the hypothesis.

**Table 7:** Uralic languages: Proportion of the nine verb pairs that use causativization.

North Saami	0.78
Kildin Saami	0.83
Finnish	0.89
Estonian	0.72
Erzya Mordvin	0.50
Mari	0.61
Udmurt	0.44
Hungarian	0.67
Mansi	0.61
Khanty	0.44
Tundra Nenets	0.78

*Languages are ordered from west to east (Hungarian is placed with the eastern languages where it originated)*.

*Hungarian has a fairly high level of causativization, the reasons for which are not examined here. Hungarian has not undergone a major spread; it moved from southern Siberia to central Europe by migration, keeping its language (and apparently its ethnic and language identity) through several centuries as an enclave in a Turkic confederation and then in the Iranian-speaking western steppe population of the post-Roman centuries*.

### Indo-European

The Indo-European family has a long history of spreads of types that should not favor symbiosis: expansions of state and imperial languages, spreads of written languages, and spreads driven by economic, technological, and/or political advantage (the earliest Indo-European spreads must have been of these latter types: see Mallory, 1989; Mallory and Adams, 1997; Anthony, 2007). The very earliest spreads, which brought the Anatolian languages (Hittite and its sisters) to what is now Turkey and the ancestors of at least Greek, Latin, and the Celtic languages to Europe, may have been migrations with formation of local outposts (Anthony, 2007) that only later grew by language shift, as was happening with Latin in early historical times; or they may have begun with invasion, conquest, and wholescale language and culture replacement in southeastern Europe (Parpola, 2012). The migration-and-outpost scenario could have produced occasional local cases of symbiosis, but more probably the outpost languages were economically prestigious and remained discrete. The invasion scenario is unlikely to have produced symbiosis.

What is striking about Indo-European is its low overall frequency of causativization (Table 8); the European cluster of low causativization in Figure 3 is mostly Indo-European languages. For the modern languages the structural reason for this is that their most common kind of pairing derives the non-causal from the causal by reflexivization (see again Table 1). Reflexivization is a post-classical development: absent from Greek, beginning to occur in Latin, halfway developed in Old Church Slavic (ninth century), and evidently it spread between early Romance, Germanic, and Slavic by calquing^6^.

**Table 8:** Indo-European: Proportion of the nine verb pairs that use causativization.

Latin	0.14
Albanian	0.11
Greek (modern)	0.22
W. Armenian	0.83
Germanic mean	0.08
Romance mean	0.26
Slavic mean	0.11
Baltic mean	0.53
Indo-Iranian:	
Kurdish	0.28
Ossetic	0.38
Persian	0.78
Pashto	0.78
Waigali	0.40 (Only five pairs found)
Palula	1.00
Hindi	1.00

*Ordering of Indo-Iranian is west to east*.

Table 8 shows proportions of causativization in some Indo-European languages and branches. Differences within and beween European branches have no obvious cause (they have not been studied closely for this survey). Comparison across the whole family reveals three general principles. First, contact with causativizing languages can increase causativization; the clear example is Western Armenian, with Turkish and Persian contact effects^7^. Second, light verb constructions, common in Iranian languages, lower the frequency of causativization. An example of a light verb construction is Tajik *xušk šudan* (dry become) ‘dry out, dry up, get dry’: *xušk kardan* (dry make) ‘dry off, dry (something)’; or English *fall asleep, go to sleep: put to sleep* or *catch (on) fire: set on fire*. These consist of an element with lexical meaning (*xušk* ‘dry’, *(a)sleep, on fire/afire*) and an auxiliary which contributes little lexical meaning but carries tense and agreement and determines the syntactic valence of the construction. Third, causativization levels are high in the Indo-Iranian branch, especially in its eastern representatives. This branch spread rapidly across the entire steppe about 4,000 years ago, propelled by development of metallurgy and metalworking in the Ural area and military advances including chariot technology. Speakers of early Indo-Iranian came to dominate, and finally absorbed, the the western Central Asian oasis civilizations of the Bactria-Margiana Archaeological Complex (Hiebert, 1994; Witzel, 2003), and the entire branch shows contact effects from a Dravidian or Dravidian-like language (the Dravidian family is indigenous to India) usually attributed to that episode. The Indic branch shows further contact effects from Dravidian. The Dravidian languages have high proportions of causativization, and it is plausible, though far from proven, that the Indo-Iranian high causativization results from these contacts. Whether any of these contacts could have produced symbiosis is a different question. Military conquest (as across the steppe) and economic dominance (as in Central Asia and later in northwestern India) usually do not, but substrata can, and certainly the deep intermingling of Indo-European and Dravidian-like or Indic-like myth and religion in Vedic Sanskrit suggests something like symbiosis^8^.

Therefore it is at least possible that the high proportions of causativization in Indo-Iranian result from symbiosis. If not symbiosis, they may result from ordinary close contact involving calquing. Western Central Asia is desert and sparsely populated–except for the oasis cities, which have large and dense populations, and were the main target of Indo-Iranian dominance. Therefore the Indo-Iranian spread to the cities was a language spread through a dense population.

### Uto-Aztecan

The Uto-Aztecan family, about 5,000 years old, ranges north-south from Shoshoni in the northern U.S. Great Basin to Nahuatl varieties throughout Mexico and an outlier in Pipil (Nicaragua, a former Aztec garrison)^9^. The family probably originated in or near Mexico, i.e. in the southern part of its range, and spread northward with or in advance of the northward advance of agriculture. Much later came the Aztec imperial spread. Daughter languages are spoken mostly by agriculturalists or (in the Great Basin) hunter-gatherers focusing on plant-based and especially seed-grinding subsistence. The two major spreads in the family are the spread of Nahuatl with the Aztec empire and the Spanish conquest (which used classical Nahuatl as official contact language), and the spread of the Numic branch through the Great Basin after a severe drought in the middle ages destroyed the early agricultural economy there.

A small sample of Uto-Aztecan languages (Table 9) gives some support to the correlation of causativization with symbiosis, with mobility and large spreads implying symbiosis. Tümpisa Shoshone, with the highest proportion of causativization, represents the highly mobile and sparse populations of the Great Basin which gave Hill (2001a) (drawing on work on Shoshoni by Wick Miller) her example of a society without stable groups of age mates and hence with minimal dialect identity. The others are settled agriculturalists; the Tohono O'odham were partly transhumant between summer and winter water sources (the transhumant population, inhabiting the driest part of the range, gave Hill her example of contingent access to resources and her documentation of variability in such populations).

**Table 9:** Uto-Aztecan languages: Proportion of the nine verb pairs that use causativization.

Tümpisa Shoshone	0.89	
Hopi	0.67	
Tohono O'odham	0.38	
Raramuri	0.78	
Huastec Nahuatl	0.60	0.75 (different analyses)

*Languages are listed from north to south*.

### Austronesian

The widespread Austronesian family originated on or near Taiwan some 6,000 years ago and spread through Island Southeast Asia and thence to near and far Oceania^10^. The spread to New Guinea and nearby islands involved coastal or offshore settlement and usually intensive contact and intermarriage as indicated by grammatical and lexical influence and genetic evidence. The spread to Micronesia and Polynesia involved colonization of previously uninhabited islands. As a result of this long history of migration the family is very large, with about 1,000 daughter languages. The eight languages in Table 10, representing all the Austronesian languages in my database, are a grossly inadequate sample of this diversity, but they cover the geographical range and some of the branches. They give some support to the hypothesis. High proportions might be expected in languages of Island Southeast Asia, where pre-Austronesian populations were absorbed in the early stages of spreading, populations are dense, and there is a history of statehood, which makes changing alliances and oscillating dominance plausible. In New Guinea and the nearby large islands, Austronesian languages colonized coastal areas, occupied a maritime economic niche, and interacted and intermarried with indigenous horticulturalists. The outcome is sometimes linguistically mixed households with multilingualism beginning in childhood, and grammatical convergence, but languages that remain discrete because they are associated with descent groups. If the situation described by Ross (1996) for north coastal New Guinea is at all common, the distinction of ethnic and inter-ethnic language and the different directions of phonological and lexicosemantic influence show that the languages are ideologically distinct and not sociolinguistically neutral. Symbiosis should not occur in such situations and the proportion of causativization should not be high. In remote Oceania, where languages mostly occupy small islands that do not foster diversity and offer few day-to-day contacts with other languages, symbiosis should not be common and causativization rates should not be high. In Table 10, the highest proportions are indeed found in Island Southeast Asia (Malay, Acehnese) and lower proportions are found elsewhere, supporting the hypothesis or at least not undermining it, but a much larger survey and community-specific accounts of sociolinguistics are needed to draw any firm conclusions. Causativization, and specific causative morphology, are ancestral in Austronesian, and here it is the retention of an attractor state that is relevant. Retention rates are lower in places where symbiosis is unlikely to have occurred, higher where it might have occurred.

**Table 10:** Austronesian languages: Proportions of the nine verb pairs that use causativization, and broad locations.

Paiwan	0.61	(Taiwan)
Malay	0.78	(Island Southeast Asia)
Acehnese	1.00	(Island Southeast Asia)
Javanese	0.60	(Island Southeast Asia)
Tolai	0.67	(Coastal New Guinea)
Tawala	0.50	(Coastal New Guinea)
Drehu	0.57	(Remote Oceania)
Samoan	0.71	(Remote Oceania)

*Languages are listed by increasing distance from the homeland*.

### The balkan sprachbund

The Balkan Sprachbund, or Balkan language area, in the southern part of the Balkan Peninsula, is the exception that proves the rule. The languages of the Sprachbund are Greek, Albanian, Macedonian, Bulgarian, southeastern (Torlak) Serbian, Arumanian (Balkan Romanian), and Romani; Turkish has been present for several centuries but does not participate in the Sprachbund. The Sprachbund is a textbook case of a linguistic area involving contact, multilingualism, and grammatical convergence^11^. Causativization is low in the Balkan Sprachbund, not appreciably different from the rest of western Europe. There has been a good deal of lexical borrowing, extensive grammatical convergence, but no selection of the attractors covered here^12^. The evident reason is that Balkan sociolinguistics is quite different from symbiosis. There is multilingualism beginning in childhood, clear language identity, language discreteness, and low tolerance for mixing and code switching. All of the languages except for Romani are national languages with written standards that further inhibit selection and mixture (though Arumanian and Torlak Serbian are quite different from the national standards). Symbiosis and selection are not expected in this situation and they have apparently not occurred in the Balkan Sprachbund.

## Discussion

Non-linguistic causation, in the domain studied here, is evidently for real, but it is not a simple cause-and-effect matter. We need a three-factor model. First, alignment with event-structure semantics and the ready availability of sources of causativizing morphemes make causativization a potential attractor. Second, the sociolinguistics of symbiosis lets selection operate. Third, the right combination of environmental and sociolinguistic conditions lets selected variants be propagated and take root. The environmental factors include deserts and high latitudes, and it should be emphasized again that the actual cause is not these geophysical environments but the sparse populations they host.

The *m-T* and *n-m* pronoun patterns used as introductory illustration have striking geographical distributions: well attested in one macrocontinent and rare elsewhere. Causativization is less black-and-white, found to appreciable extents everywhere except Europe, and it is more frequent worldwide. Some of the difference may be in how the two are measured (causativization is sought over a larger wordlist than the basic first and second person pronouns), but the main factor must be ease of selection: borrowing of pronouns is generally inhibited, but pattern copying of verb derivational structure is more readily tolerated (as shown by accommodation of derivational types to those of neighboring languages, discussed for Western Armenian and Romani).

Language families vary in their mean frequencies of causativization, and most of that variation reflects not the non-linguistic causes described here but relatively stable family traits. Therefore the effect of symbiosis and the relevant environmental factors is to raise or lower proportions of causativization relative to family means. There is no absolute threshold above which symbiosis can be confidently posited and below which it cannot.

Symbiosis is a product of intense contact, but not all intense contact produces symbiosis. The Balkan Sprachbund is the clearest case of intense contact without symbiosis. Other areas known to have language identity, linguistic discreteness, and grammatical but not lexical convergence include northern Australia and much of Amazonia, where societies and languages are smaller and languages are mostly unwritten but the sociolinguistics and striking combination of shared grammar and discrete lexicons are also present. Another kind of contact situation without symbiosis is asymmetrical dominance, where one language is more widely used or valued than another (for reasons such as political dominance, national language used in education vs. minority language restricted to home use, economic usefulness, inter-ethnic language, educational policy, etc.), a situation that often leads to language shift and drives the non-dominant language into extinction. In the great variety of language contact scenarios and sociolinguistic situations, symbiosis is not particularly common, but the results presented here show that it does occur and can be identified with reasonable reliability, even prehistorically.

Such are the non-linguistic causes that nudge languages toward greater use of causativization. Given these promising results, work about to begin will survey more families, more of their daughter languages, and more structural variables, and will cover sociolinguistic, ethnographic, and demographic factors in more depth.

## Author contributions

JN designed the study, did some of the data collection and analysis, carried out most of the analysis, and wrote the article.

### Conflict of interest statement

The author declares that the research was conducted in the absence of any commercial or financial relationships that could be construed as a potential conflict of interest.

## Footnotes

^1^A case of iconicity; see e.g., (Haiman, 1985) and much other work.

^2^Sources for the historical and sociolinguistic description in the next paragraphs include (Lavrov, 1953; Volkova, 1967; Wixman, 1980; Aglarov, 1988, 1994, 2002; Nichols, 2005, 2016; Karpov and Kapustina, 2011; Dobrushina, 2013). Here and below, for each section I cite sources used and a few well-known overviews, selecting from a very large literature on each topic.

^3^They adopted the name of an important pre-Hunnish nomadic society from the eastern steppe. The ethnonym had in turn been taken on by the Avars who ruled central Europe from the 6th to 8th centuries, attacking Byzantium and invading the Balkan peninsula. Apart from the ethnonym there is no connection between the Caucasian Avars and the other two groups.

^4^Sources on the history and sociolinguistics for this section include (Krader, 1963; Barfield, 1989; Chernykh, 1992, 2009; Khazanov, 1994; Janhunen, 1996, 2008, 2012; Pulleyblank, 2000; Schönig, 2003; Anthony, 2007; Kohl, 2007; Di Cosmo et al., 2009; Hanks, 2010; Golden, 2011; Frachetti, 2012; Werner, 2014; Vovin et al., 2016; Nichols and Rhodes, 2017).

^5^Map of modern distributions: https://en.wikipedia.org/wiki/Uralic_languages#/media/File:Linguistic_map_of_the_Uralic_languages_(en).png. This is the visually clearest map I have found, but the subgroupings listed are not all correct. Current classification: http://www.helsinki.fi/~tasalmin/fu.html Map showing branch homelands: http://www.helsinki.fi/~tasalmin/Uralic.jpg. Other sources for this section: (Sinor, 1988, 1990; Napol'skix, 1997; Abondolo, 1998; Anthony, 2007; Grünthal and Petri, 2012; Holopainen, 2017).

^6^For general aspects of verb root and stem structure in Indo-European see e.g., (Rix, 2001; Jasanoff, 2003; Fortson, 2010).

^7^It can also retard loss of causativization: Romani varieties in Europe and nearby have generally lost the inherited Indic causative morphology except for varieties in contact with Turkic (Adamou, 2012; E. Adamou p.c.).

^8^For the Indo-Iranian takeover of the Central Asian civilizations see (Witzel, 2003; Anthony, 2007; Frachetti, 2008; Kuzmina, 2008); for the civilization (Hiebert, 1994).

^9^Sources for this section: (Fowler, 1972; Miller, 1983; Madsen and Rhode, 1994; Hill, 2001b, 2010; Kemp et al., 2010; Golla, 2011; Merrill, 2012).

^10^See e.g., (Pawley and Ross, 1993; Friedlaender et al., 2008; Ross et al., 2008; Blust, 2009; Kirch, 2010; Donohue and Denham, 2012; Bellwood, 2017).

^11^Overviews of the Balkan area include (Joseph, 1983; Thomason, 2001; Aronson, 2008; Friedman, 2011).

^12^A prominent thread in Balkanist literature describes such Balkan traits as loss of case inflection and some affixal tense-mood inflection and their replacement by clitics and particles as an increase in analyticity and thereby in transparency, a change that also favors convergence by making grammatical formatives easily calquable (e.g., Lindstedt, 2000). This is a form of simplification and a favored outcome of contact-induced change, but favored outcomes is a broader notion than attractor as defined here.

## References

[B1] Abondolo D. (ed.). (1998). The Uralic Languages. London; New York, NY: Routledge.

[B2] Adamou E. (ed.). (2012). Verb morphologies in contact: Evidence from the Balkan area, in Morphologies in Contact, eds Martine Vanhove T. S. Urdze A. Otsuka H. (Berlin: De Gruyter), 143–162.

[B3] Aglarov M. A. (1988). Sel”skaja obschina v Nagornom Dagestane v XVII-nachale XIX v. Moscow: Nauka.

[B4] Aglarov M. A. (1994). Andi, in Encyclopedia of World Cultures, VI: Russia and Eurasia/China, eds Friedrich P. Diamond N. (Boston: G. K. Hall & Co), 23–27.

[B5] Aglarov M. A. (2002). Andijcy: Istoriko-ètnografičeskoe issledovanie. Maxachkala: Jupiter.

[B6] Anthony D. W. (2007). The Horse, the Wheel, and Language: How Bronze Age Riders from the Eurasian Steppes Shaped the Modern World. Princeton, NJ: Princeton University Press.

[B7] Aronson H. I. (2008). The Balkan Linguistic League, “Orientalism,” and Linguistic Typology. Ann Arbor, MI: Beech Stave.

[B8] Bakker P. Mous M. (eds.). (1994). Mixed Languages: 15 Case Studies in Language Intertwining. Amsterdam: IFOTT.

[B9] Barfield T. (1989). The Perilous Frontier: Nomadic Empires and China, 221 BC to AD 1757. London: Blackwell.

[B10] Bellwood P. S. (2017). First Islanders: Prehistory and Human Migration in Island Southeast Asia. Hoboken: Wiley.

[B11] Blust R. A. (2009). The Austronesian Languages. Canberra: Research School of Pacific and Asian Studies, Australian National University Available online at: http://pacling.anu.edu.au/materials/Blust2013Austronesian.pdf

[B12] Chernykh E. N. (1992). Ancient Metallurgy in the USSR: The Early Metal Age. New York, NY: Cambridge University Press.

[B13] Chernykh E. N. (2009). Formation of the Eurasian steppe belt cultures: Viewed through the lens of archaeometallurgy and radiocarbon dating, in Social Complexity in Prehistoric Eurasia: Monuments, Metals, and Mobility, eds Hanks B. K. Linduff K. M. (Cambridge: Cambridge University Press), 115–145.

[B14] Creissels D. (2014). P-lability and radical P alignment. Linguistics 52, 911–944. 10.1515/ling-2014-0012

[B15] Dahl Ö. (2004). The Growth and Maintenance of Linguistic Complexity. Amsterdam: Benjamins.

[B16] Di Cosmo N. Fank A. J. Golden P. B. (eds). (2009). The Cambridge History of Inner Asia: The Chinggisid Age. Cambridge: Cambridge University Press.

[B17] Dobrushina N. (2013). How to study multilingualism of the past: Investigating traditional contact situations in Daghestan. J. Sociolinguist. 17, 376–393. 10.1111/josl.12041

[B18] Donohue M. Denham T. (2012). Lapita and proto-oceanic: thinking outside the pot? J. Pac. History 47, 443–457. 10.1080/00223344.2012.742609

[B19] Fagyal Z. Samarth S. Escobar A. M. Gasser L. Lakkaraju K. (2010). Centers and peripheries: Network role in language change. Lingua 120 2061–2079. 10.1016/j.lingua.2010.02.001

[B20] Forker D. (2013). A Grammar of Hinuq. Berlin: de Gruyter Mouton.

[B21] Fortson B. W. I. V. (2010). Indo-European Language and Culture: An introduction. Malden, MA; Oxford: Blackwell.

[B22] Fowler C. S. (1972). Some ecological clues to Proto-Numic homelands, in Desert Research Institute Publications in the Social Sciences, 8. Great Basin Cultural Ecology: A Symposium (Reno: Desert Research Institute), 105–121.

[B23] Frachetti M. D. (2008). Pastoralist Landscapes and Social Interaction in Bronze Age Eurasia. Berkeley, CA; Los Angeles, CA: University of California Press.

[B24] Frachetti M. D. (2012). Multiregional emergence of mobile pastoralism and nonuniform institutional complexity across Eurasia. Curr. Anthropol. 53, 2–38. 10.1086/663692

[B25] Friedlaender J. S. Friedlaender F. R. Reed F. A. Kidd K. K. Kidd J. R. Chambers G. K.. (2008). The genetic structure of Pacific Islanders. PLoS Genet. 4:e19. 10.1371/journal.pgen.0040019 18208337 PMC2211537

[B26] Friedman V. A. (2011). The Balkan languages and Balkan linguistics. Annu. Rev. Anthropol. 40, 275–291. 10.1146/annurev-anthro-081309-145932

[B27] Golden P. (2011). Central Asia in World History. Oxford: Oxford University Press.

[B28] Golla V. (2011). California Indian Languages. Berkeley, CA; Los Angeles, CA: University of California Press.

[B29] Grünthal R. Petri K. (2012). eds. A Linguistic Map of Prehistoric Northern Europe. Helsinki: Société Finno-Ougrienne.

[B30] Grünthal R. Nichols J. (in press). Transitivizing/detransitivizing typology language family history. Lingua Posnaniensis.

[B31] Haiman J. (1985). Natural syntax: Iconicity and erosion. Cambridge: Cambridge University Press.

[B32] Hanks B. (2010). Archaeology of the eurasian steppes and mongolia. Annu. Rev. Anthropol. 39, 469–486. 10.1146/annurev.anthro.012809.105110

[B33] Hiebert F. T. (1994). Origins of the Bronze Age Oasis Civilization in Central Asia. Cambridge, MA: Peabody Museum of Archaeology and Ethnology, Harvard University.

[B34] Hill J. H. (2001a). Languages on the land, in Language, Archaeology, and History, ed Terrell J. (Westport, CT: Bergin & Garvey), 257–282.

[B35] Hill J. H. (2001b). Proto-Uto-aztecan: a community of cultivators in central America? Am. Anthropol. 103, 913–934. 10.1525/aa.2001.103.4.913

[B36] Hill J. H. (2010). New evidence for a Mesoamerican homeland for Proto-Uto-Aztecan. Proc. Natl. Acad. Sci. U.S.A. 107, E33. 10.1073/pnas.0914473107 20231477 PMC2841890

[B37] Holopainen S. (2017). Indo-Iranian loanwords in Uralic. Ph.D dissertation, University of Helsinki.

[B38] Janhunen J. (1990). Material on Manchurian Khamnigan Mongol. Helsinki: Finno-Ugrian Society.

[B39] Janhunen J. (1991). Material on Manchurian Khamnigan Evenki. Helsinki: Finno-Ugric Society.

[B40] Janhunen J. (1996). Manchuria: An Ethnic History. Helsinki: Suomalais-Ugrilainen Seura.

[B41] Janhunen J. (2002). On the chronology of the Ainu ethnic complex. Bull. Hokkaido Mus. Northern Peop. 11, 1–20.

[B42] Janhunen J. (2005). Khamnigan Mongol. Munich: Lincom Europa.

[B43] Janhunen J. (2008). Mongolic as an expansive language family, in Past and Present Dynamics: The Great Mongolian State, ed Kurebito T. Tokyo: Tokyo University of Foreign Studies, Research Institute for Languages and Cultures of Asia and Africa, 127–137.

[B44] Janhunen J. (2012). The expansion of Tungusic as an ethnic and linguistic process, in Recent Advances in Tungusic Linguistics, eds Malchukov A. L. Whaley L. J. (Harrassowitz: Wiesbaden), 5–16.

[B45] Jasanoff J. H. (2003). Hittite and the Indo-European Verb. Oxford: Oxford University Press.

[B46] Joseph B. (1983). The Synchrony and Diachrony of the Balkan Infinitive: A Study in Areal, General, and Historical Linguistics. Cambridge: Cambridge University Press.

[B47] Karpov J. J. Kapustina E. L. (2011). Gorcy posle gor: Migracionnye processy v Dagestane v XX-nachale XXI vv.: ix social”nye i etnokul”turnye posledstvija i perspektivy. St. Petersburg: Rossijskaja AN, Muzej antropologii i etnografii.

[B48] Kemp B. M. González-Oliver A. Malhi R. S. Monroe C. Schroeder K. B. McDonough J.. (2010). Evaluating the farming/language dispersal hypothesis with genetic variation exhibited by populations in the Southwest and Mesoamerica. Proc. Natl. Acad. Sci. U.S.A. 107, 6759–6764. 10.1073/pnas.0905753107 20351276 PMC2872417

[B49] Khazanov A. M. (1994). Nomads and the Outside World. Madison, WI: University of Wisconsin Press.

[B50] Kirch P. V. (2010). The peopling of the Pacific: A holistic anthropological perspective. Annu. Rev. Anthropol. 39, 131–148. 10.1146/annurev.anthro.012809.104936

[B51] Kohl P. L. (eds.). (2007). The Making of Bronze Age Eurasia. Camridge: Cambridge University Press.

[B52] Krader L. (1963). Social Organization of the Mongol-Turkic Pastoral Nomads. The Hague: Mouton.

[B53] Kuzmina E. E. (2008). The Prehistory of the Silk Road. Philadelphia, PA: University of Pennsylvania Press.

[B54] Lavrov L. I. (1953). Nekotorye itogi raboty Dagestanskoj èkspedicii 1950-52 gg. Kratkie soobsčenija Instituta Ètnografii 19, 3–7.

[B55] Lindstedt J. (2000). Linguistic balkanization: contact-induced change by mutual reinforcement, in Languages in Contact, eds Gilbers D. G. Nerbonne J. Schaeken J. (Amsterdam; Atlanta: Rodopi), 231–246.

[B56] Madsen D. Rhode D. (1994). Across the West: Human Population Movement and the Expansion of the Numa. Salt Lake City, UT: University of Utah Press.

[B57] Mallory J. P. (1989). In Search of the Indo-Europeans: Language, Archaeology, and Myth. New York, NY: Thames & Hudson.

[B58] Mallory J. P. Adams D. Q. (1997). Encyclopedia of Indo-European Culture. London: Fitzroy Dearborn.

[B59] Meakins F. (2013). Mixed Languages, in Mixed Languages: A Comprehensive Guide, eds Bakker P. Matras Y. (Berlin: Mouton de Gruyter), 159–228.

[B60] Merrill W. L. (2012). The historical linguistics of Uto-Aztecan agriculture. Anthropol. Linguistics 54, 203–260.

[B61] Miller W. R. (1983). Uto-Aztecan languages, in Handbook of North American Indians, Vol. 10, ed Ortiz A. (Washington, DC: Smithsonian Institution), 113–124.

[B62] Milroy J. Milroy L. (1985). Linguistic change, social network, and speaker innovation. J. Linguist. 21, 339–384.

[B63] Milroy L. Milroy J. (1992). Social network and social class: Toward an integrated sociolinguistic mode. Lang. Soc. 21, 1–6.

[B64] Napol'skix V. V. (1997). Vvedenie v istoričeskuju uralistiku. Izhevsk: RAN: Ural”skoe otdelenie: Udmurtskij istitut istorii, jazyka i literatury.

[B65] Nichols J. (2005). The origin of the Chechen and Ingush: a study in alpine linguistic and ethnic geography. Anthropol. Linguist. 46, 129–155.

[B66] Nichols J. (2011). Causativization and contact in Nakh-Daghestanian, in Proceedings of the 37th Annual Meeting: Special Session on Languages of the Caucasus, eds Cathcart C. Kang S. Sandy C. S. (Berkeley, CA: Berkeley Linguistics Society), 68–80.

[B67] Nichols J. (2012a). Selection for *m: T* pronominals in Eurasia, in Copies vs. Cognates in Bound Morphology, eds Johanson L. Robbeets M. (Leiden: Brill), 47–70.

[B68] Nichols J. (2012b). The history of an attractor state: Adventitious *m* in Nakh-Daghestanian, in Per Urales ad Orientem: Iter Polyphonicum Multilingue (Festschrift for Juha Janhunen), eds Hyytiäinen T. Jalava L. Saarikivi J. Sandman E. (Helsinki: SUST), 261–278.

[B69] Nichols J. (2013). The origin and evolution of case-suppletive pronouns: Eurasian evidence, in Languages across Boundaries: Studies in Memory of Anna Siewierska, eds Bakker D. Haspelmath M. (Berlin: De Gruyter Mouton), 313–345.

[B70] Nichols J. (2016). Complex edges, transparent frontiers: Grammatical complexity and language spreads, in Complexity, Isolation, and Variation, eds Baechler R. Seiler G. (Berlin: de Gruyter), 117–137.

[B71] Nichols J. Peterson D. A. (2013a). M-T pronouns. eds Dryer M. Haspelmath M. The World Atlas of Language Structures Available online at: http://wals.info/chapter/136

[B72] Nichols J. Peterson D. A. (2013b). N-M Pronouns. eds Dryer M. Haspelmath M. The World Atlas of Language Structures Available online at: http://wals.info/chapter/137

[B73] Nichols J. Peterson D. A. Barnes J. (2004). Transitivizing and detransitivizing languages. Linguist. Typolo. 8,m 2149–2211.

[B74] Nichols J. Rhodes R. A. (2017). Vectors of Language Spread at the Central Steppe Periphery: Finno-Ugric as Catalyst Language. British Archaeological Reports (International Series), special issue edited by Rune Iverson & Guus Kroonen.

[B75] Parpola A. (eds.). (2012). Formation of the Indo-European and Uralic (Finno-Ugric) language families in the light of archaeology: revised and integrated “total” correlations. Grünthal and Kallio 119–184.

[B76] Pawley A. K. Ross M. D. (1993). Austronesian historical linguistics and culture history. Annu. Rev. Anthropol. 22, 425–459.

[B77] Pulleyblank E. G. (2000). Tribal confederations of uncertain identity, in History of the Turkic Peoples in the Pre-Islamic Period, ed Roemer H. R. (Berlin: Klaus Schwarz Verlag), 52–101.

[B78] Rix H. (eds.). (2001). Lexikon der Indogermanischen Verben. Die Wurzeln und ihre Primärstammbildungen. Wiesbaden: Reichert.

[B79] Ross M. D. (1996). Contact-induced change and the comparative method: cases from Papua New Guinea, in The Comparative Method Reviewed, eds Durie M. Ross M. D. (New York, NY: Oxford University Press).

[B80] Ross M. D. Pawley A. K. Osmond M. (eds.). (2008). The Lexicon of Proto Oceanic. Canberra, ACT: Research School of Pacific and Asian Studies, Australian National University.

[B81] Schönig C. (2003). Turko-Mongolic Relations in The Mongolic Languages, ed Janhunen J. (London: Routledge), 403–419.

[B82] Sinor D. (ed.). (1990). The Cambridge History of Early Inner Asia. Cambridge: Cambridge University Press.

[B83] Sinor D. (ed.). (1988). The Uralic Languages: Description, History, and Foreign Influences. Leiden: Brill.

[B84] Thomason S. G. (2001). Language Contact: An Introduction. Washington, DC: Georgetown University Press.

[B85] Trudgill P. (2011). Sociolinguistic Typology: Social Determinants of Linguistic Structure and Complexity. Oxford: Oxford University Press.

[B86] Volkova N. G. (1967). Voprosy dvujazyčija na Severnom Kavkaze. Sovetskaja etnografija 1967, 27–40.

[B87] Vovin A. Vajda E. de la Vaissière É. (2016). Who were the ^*^Kjet (羯) and what language did they speak? J. Asiatiq. 304, 125–144.

[B88] Werner H. (2014). Die Jenissejer unter den frühen Völkern Zentralasiens. Munich: Lincom Europa.

[B89] Witzel M. (2003). Linguistic Evidence for Cultural Exchange in Prehistoric Western Central Asia. (Sino-Platonic Papers 129.) Philadelphia, PA: Department of East Asian Language and Civilization, University of Pennsylvania.

[B90] Wixman R. (1980). Language Aspects of Ethnic Patterns and Processes in the North Caucasus. Chicago, IL: University of Chicago.

[B91] Yu W. (2011). A study of the Mongol Khamnigan Spoken in Northeastern Mongolia. Seoul: Seoul National University Press.

[B92] Zgusta R. (2015). Peoples of Northeast Asia through Time: Precolonial Ethnic and Cultural Processes along the Coast between Hokkaido and the Bering Strait. Leiden: Brill.

